# Magnet and button battery ingestion in children: multicentre observational study of management and outcomes

**DOI:** 10.1093/bjsopen/zrac056

**Published:** 2022-06-03

**Authors:** Paediatric Surgery Trainee Research Network, Jonathan J. Neville, Rachel Harwood, George S. Bethell, Hannah Rhodes, Felicity Arthur, Mary P. Eastwood, Sesi Hotonu, Lucinda Tullie, Nigel J. Hall, R. Coulson, S. Lawther, K. Burns, C. S. Chacon, T. Boam, S. A. Clarke, J. Hallett, N. Valliant, T. Hemanshoo, A. Tahira, E. Decker, T. Ahmed, J. Cave, A. Ram, M. Shenoy, M. John, M. Wyn, L. Wilkins, B. Allin, A. Fagelnor, G. Bough, A. T. Mohd-Amin, R. Trenear

## Abstract

**Background:**

Magnets and button batteries (BBs) are dangerous ingested foreign bodies in children. The scale and consequences of this public health issue in the UK are unknown. This study aims to report the current management strategies and outcomes associated with paediatric magnet and BB ingestion in the UK.

**Methods:**

This multicentre, retrospective observational study involved 13 UK tertiary paediatric surgery centres. Children aged under 17 years, admitted between 1 October 2019 and 30 September 2020, following magnet, or BB ingestion were included. Demographics, investigations, management, and complications were recorded.

**Results:**

In total, 263 patients were identified, comprising 146 (55.5 per cent) magnet, 112 (42.6 per cent) BB, and 5 (1.9 per cent) mixed magnet BB ingestions. Median (interquartile range) age was 4.8 (2.0–9.1) years and 47.5 per cent were female. In the magnet group, 38 (26.0 per cent) children swallowed single magnets, 3 of whom underwent endoscopic retrieval for oesophageal or gastric impaction. Of the 108 (74.0 per cent) children who swallowed multiple magnets, 51 (47.2 per cent) required endoscopic or surgical intervention, predominantly for failure of magnets to progress on serial imaging. Bowel perforations occurred in 10 children (9.3 per cent). Younger age and ingestion of greater numbers of multiple magnets were independently associated with surgery. BB ingestion caused morbidity in 14 children (12.5 per cent) and life-threatening injuries in two (1.8 per cent); the majority were caused by oesophageal BBs (64.3 per cent).

**Conclusion:**

Multiple magnet and BB ingestions are associated with significant morbidity. Action must be taken at an international level to regulate the sale of magnets and BBs, and to raise awareness of the risks that these objects pose to children.

## Teaser

This is the largest study of paediatric magnet and button battery ingestions in the UK to date, highlighting an association with significant morbidity. Increased regulation on the sale of magnets and button batteries is required. Parents and healthcare professionals must be aware of the threat that these objects pose to children when ingested.

## Introduction

Foreign body ingestion commonly occurs in the paediatric population. Young children inadvertently swallow objects found while exploring their environment, and older children may purposefully or accidentally swallow objects found in the home^1^. Biologically inert objects typically pass through the gastrointestinal tract without complication^2^. However, some foreign bodies can interact with gastrointestinal tissues, causing significant morbidity^3^.

A single magnetic object may spontaneously pass through the gastrointestinal tract, but multiple magnets can require endoscopic or surgical removal to prevent or treat complications of ingestion^4–6^. Multiple magnets can attract one another from different locations within the gastrointestinal tract, causing the failure of the magnets to progress, luminal obstruction, and localized pressure necrosis resulting in fistula formation and perforation^6,7^.

Ingested button batteries (BBs) may become impacted in the oesophagus of young children, where they have the potential to cause perforation, mediastinitis, fistulae, and strictures^8^. Caustic burns from ingested BBs cause coagulative necrosis within 15 min, and maximal damage occurs within 12 h^9^. Delayed complications have been described, with fatal aorto-oesophageal fistulae presenting up to 3 weeks after ingestion^10^. These dangers are compounded by the fact that ingestions may be unwitnessed or go unrecognized. Early symptoms of hazardous foreign body ingestions are non-specific, and children may present after irreversible damage has begun to occur^1,4^.

The incidence of paediatric magnet and BB ingestions have increased globally in the last decade^1,6,11,12^ due to the increased availability of small, powerful rare-earth neodymium magnets in toys, and the widespread presence of larger, higher voltage lithium-ion BBs in electronic devices. This study aims to describe the current management and associated morbidity of such ingestions across the UK. These contemporary multicentre data will enable the development of preventative strategies and public health policy for both BBs and magnets. Furthermore, they will inform clinicians on the current practice for this relatively novel problem.

## Methods

### Study design and participants

This was a multicentre retrospective study conducted by the UK-based Paediatric Surgery Trainee Research Network (PSTRN). Results are reported in accordance with STROBE guidelines^13^.

All 28 UK centres providing tertiary paediatric surgery were eligible to participate and were approached by the PSTRN. The study was registered at each participating institution as a service evaluation. Anonymized data were retrospectively collected for children admitted to hospital, between 1 October 2019 and 30 September 2020, using a standardized proforma. Participants were identified using the ICD Diseases and Related Health Problems (ICD10-GM) code for ‘foreign body in alimentary tract’: T18. A complete search of paper and electronic case notes was performed to identify all patients admitted to hospital with the code T18. Children who had ingested magnets or BBs were then identified from this search.

All patients aged under 17 years at time of hospital admission, with the presence of at least one magnetic foreign body or BB in the gastrointestinal tract, were eligible for inclusion. The number of patients with foreign bodies of any other type were recorded, but not included in further analysis. Patients or the public were not involved in the study design. Data were extracted from patient paper and electronic case notes.

### Outcomes

Demographic information, foreign body details, type, and frequency of diagnostic imaging studies, management and intervention strategies and timings, and outcome data were extracted from case notes by a member of the local clinical team. The anatomical location of the foreign body on admission was determined by the treating clinician. Subsequent locations were determined either by radiology, endoscopy, or at surgery. Intervention strategies were defined as conservative (‘watch and wait’, or medications, including proton pump inhibitors (PPIs), laxatives, and enemas), endoscopic, or surgical. ‘Initial treatment’ refers to the first management strategy used. For example, initial conservative management includes planning to repeat a radiograph or monitor the child for signs of clinical deterioration before electing to undertake endoscopic or surgical management. Successful conservative management occurred if no further interventional treatments (endoscopic or surgical) were required. Treatment success was defined as the complete passage or removal of all foreign bodies. A previous diagnosis of a behavioural disorder such as autism spectrum disorder (ASD) or attention-deficit disorder (ADD) was also recorded. Psychiatric diagnoses (anxiety and depression) were not recorded.

### Statistical analysis

Data are presented as median (interquartile range (i.q.r.)) unless otherwise specified. Statistical analyses were conducted using SPSS^®^ version 27 (IBM, Armonk, New York, USA) and Prism version 7 (GraphPad Software, San Diego, California, USA). Non-parametric continuous datasets were compared using the Mann–Whitney *U* test. Categorical variables were compared using the Fisher’s exact test or chi-squared test as appropriate. Logistic regression analysis was used to identify factors associated with surgical intervention after magnet ingestion. Age and number of magnets swallowed were assessed as continuous variables. Sex and the presence of behavioural diagnoses were assessed as binomial variables with ‘female sex’ and ‘no behavioural diagnoses’ as reference groups. Data are described using OR and 95 per cent confidence intervals (c.i.). A *P* value < 0.05 was considered significant.

## Results

A total of 263 children were identified from 13 paediatric surgery centres. These included 146 magnet (55.5 per cent), 112 BB (42.6 per cent), and five mixed magnet and BB ingestions (1.9 per cent) (*Table 1*). Ten centres recorded the total number children admitted with foreign bodies in the alimentary tract (*n* = 537), of which 185 (34.5 per cent) were magnet and BB ingestions. Median (i.q.r.) age on admission was 4.8 (2.0–9.1) years and 47.5 per cent of children were female. Children who ingested BBs were younger than those who ingested magnets (3.0 *versus* 7.0 years, *P* < 0.001).

**Table 1:** Demographic features and radiological location of foreign bodies on admission.

	Single magnet ingestion (*n* = 38)	Multiple magnet ingestion (*n* = 108)	Button battery ingestion (*n* = 112)
**Age**			
<1 year	0 (0)	0 (0)	6 (5.4)
1–4 years	15 (39.5)	36 (33.3)	73 (65.2)
5–9 years	16 (42.1)	32 (29.6)	21 (18.8)
10–14 years	6 (15.8)	37 (34.3)	4 (3.6)
≥15 years	1 (2.6)	3 (2.8)	8 (7.1)
**Female**	16 (42.1)	53 (49.1)	53 (47.3)
** Initial location on imaging**			
Oesophagus	2 (5.3)	0 (0)	28 (25.0)
Stomach	20 (52.6)	49 (45.4)	65 (58.0)
Small Bowel	11 (29.0)	48 (44.4)	8 (7.1)
Large Bowel	5 (13.2)	11 (10.2)	9 (8.0)
Unknown	–	–	2 (1.8)

Values are n (%).

### Behavioural diagnoses

Twenty-one (8.0 per cent) children had a behavioural diagnosis (*Table 2*). Presence of a behavioural diagnosis did not differ significantly between the magnet and BB ingestion groups (*P* = 0.964). Those with a behavioural diagnosis were older (11.6 *versus* 4.0 years, *P* = 0.001), and ingested a significantly greater median number of BBs (2.0 *versus* 1.0, *P* = 0.007), but not magnets (2.5 *versus* 3.0, *P* = 0.718).

**Table 2:** Comparison of children with and without a behavioural diagnosis.

	Behavioural diagnosis (*n* = 21)	No behavioural diagnosis (*n* = 196)	*P*
**Age (years)**	11.6 (5.5–15.0)	4.0 (2.0–8.4)	*0*.*001*
**Female**	11 (52.4)	94 (48.0)	0.700
**Number of magnets ingested**	2.5 (1.0–4.0)	3.0 (2.0–6.0)	0.718
**Number of button batteries ingested**	2.0 (1.0–5.0)	1.0 (1.0–1.0)	*0*.*007*
**Number of interventions**	1.0 (1.0–1.0)	1.0 (1.0–1.0)	0.544
**Time to intervention one (days)**	0 (0–1.5)	0 (0–1.0)	0.875
**Duration of hospital stay (days)**	1.0 (0–3.0)	1.0 (0–3.0)	0.857
**Morbidity, n (%)**	2 (9.5)	33 (16.8)	0.387

Values are median (i.q.r.) unless otherwise indicated.

### Magnet ingestion

Confirmation of magnet ingestion was achieved by plain-film X-ray in 144 cases (98.6 per cent). Ultrasound and CT were used to diagnose one further case each. Of 146 magnet ingestions, 108 children (74.0 per cent) ingested two or more magnets. The median (i.q.r.) number of magnets ingested was 2.0 (1.0–5.0; range 1.0–76.0).

#### Ingestion of a single magnet

Conservative management was used successfully in 35 children (92.1 per cent) who swallowed a single magnet. Endoscopy was performed in the remaining cases to remove single magnets located in the oesophagus (*n* = 2), and stomach (*n* = 1). Endoscopic retrieval was used because of the large size of these magnets. The median (i.q.r.) number of plain-film X-rays performed for a single ingested magnet was 1.0 (1.0–2.0; range 1.0–5.0). No children underwent CT.

#### Ingestion of multiple magnets

**Fig. 1:**
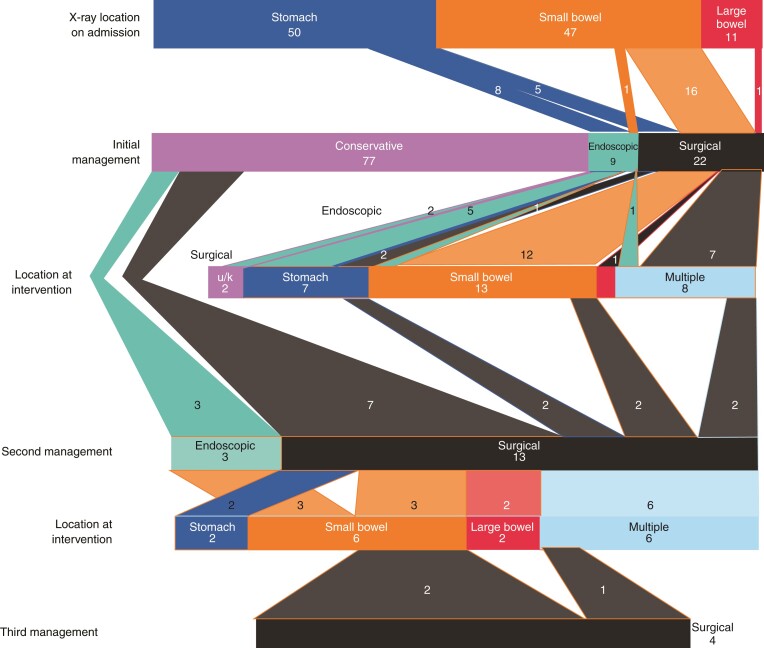
**Sankey plot depicting location and management of children admitted to hospital after ingesting multiple magnets** The suspected initial location of the magnets is shown in the first row, with the second row showing the initial intervention modality, and the third row showing the actual location of the magnets upon intervention. Initial management was endoscopic or surgical in 31 of 108 cases (28.7 per cent). Conservative management was undertaken in 77 cases and failed in 10 (13.0 per cent) cases. Overall, of the 108 children who swallowed multiple magnets, 39 (36.1 per cent) underwent surgical management. u/k, unknown.

Of the 108 children who swallowed two or more magnets, initial management was conservative in 77 (71.3 per cent), endoscopic in 9 (8.3 per cent), and surgical in 22 (20.4 per cent) (*Fig. 1*). Initial conservative management was abandoned in 10 children, who subsequently proceeded to endoscopic or surgical intervention. In these 10 patients, conservative management was undertaken for a median (i.q.r.) of 3 (1.3–3.8; range 0–14.0) days before escalation. The median (i.q.r.) time to initial intervention across all 108 children swallowing multiple magnets was 1.0 (0–2.0; range 0–14.0) days after ingestion and the median (i.q.r.) time to surgical intervention was 1.0 (0–3.0; range 0–14.0) day.

Surgery was performed in 39 of 108 (36.1 per cent) children, including 28 undergoing laparotomies, two laparoscopies, and nine unspecified surgeries. The most common indications for surgical intervention were the failure of multiple magnets to progress on serial imaging (46.1 per cent), and clinical suspicion or evidence of bowel perforation (41.0 per cent). An inability to remove the magnets endoscopically prompted surgery in 7.7 per cent of children. Bowel perforations requiring resection occurred in three cases. Seven children suffered bowel perforations that were repaired without resection. Magnets caused bowel obstruction in two cases. Three children were noted to have developed bowel fistulae. Two patients were noted to have bowel mucosal damage. No children required a stoma. The remaining 22 patients underwent surgery to remove multiple magnets, but no complications were specified.

In the multiple magnet cohort, the median (i.q.r.) number of plain-film X-rays undertaken was 3.0 (2.0–4.0; range 1.0–10.0). This was significantly higher than in the single magnet group (3.0 *versus* 1.0, *P* < 0.001). Six patients underwent CT.

#### Factors associated with operative management after multiple magnet ingestion

Predictor variables of sex, age, underlying behavioural diagnosis (ASD and ADD), and number of magnets ingested were assessed for an association with surgical intervention using univariable analysis (*Table 3*). Multivariable logistic regression demonstrated a continued significant association between increasing numbers of ingested magnets (OR 1.12; 95 per cent c.i. 1.03 to 1.24; *P* 0.022); however, age became insignificant.

**Table 3:** Characteristics of children undergoing surgical intervention *versus* no surgical intervention.

	Operative management (*n* = 35)	No operative management (*n* = 73)	*P*
**Female:male ratio**	17:18	35:38	>0.999
**Age (years)**	3 (2–12)	9 (5–12)	0.002
**Underlying behavioural diagnosis, n (%)**	3 (8.6)	5 (6.8)	>0.999
**Number of ingested magnets**	6 (4–7)	3 (2–4)	<0.001

Values are median (i.q.r.) unless otherwise indicated.

#### Complications following surgical intervention

Two (1.9 per cent) of the 108 children who swallowed multiple magnets required three laparotomies to remove the magnets and treat subsequent complications. One child ingested 16 magnets, developed gastric and duodenal perforations, and required re-look laparotomies to reassess the bowel. A further child who swallowed four magnets required a second laparotomy for adhesional bowel obstruction.

One child developed a postoperative wound infection, one child required parenteral nutrition due to exacerbation of an eating aversion, and another child was readmitted for parenteral feeding due to poor nutrition. The median (i.q.r.) duration of hospital stay was 1.0 (0–4.0; range 0–156.0) day, and patients were followed up at a median (i.q.r.) of 3.0 (1.0–4.0; range 0–10.0) months. There was no mortality in this group.

### Button battery ingestion

The median (i.q.r.) age of children who ingested BBs was 3.0 (1.2–5.0) years and the median (i.q.r.) number of BBs ingested was 1.0 (1.0–1.0; range 1.0–14.0). Of the 112 BB ingestions, 28 (25.0 per cent) were identified in the oesophagus and 65 (58.0 per cent) in the stomach on admission. Only three (2.7 per cent) were seen in the duodenum, five (4.5 per cent) in the small intestine and nine (8.0 per cent) in the large intestine. Diagnosis was made by plain X-ray in 110 (98.2 per cent) children and CT in one (0.9 per cent) case. Diagnostic modality was unspecified in one case (0.9 per cent). The median (i.q.r.) number of X-rays taken for each patient was 2.0 (1.0–3.0; range 1.0–23.0).

Conservative management was employed initially in 62 (55.4 per cent) children, including seven patients with oesophageal BBs, one of whom went on to have endoscopic retrieval the following day. Primary endoscopic retrieval was undertaken in 41 (36.6 per cent) children; 18 (43.9 per cent) BBs were located in the oesophagus, 22 (53.7 per cent) in the stomach, and one (4.9 per cent) in the rectum.

Surgical intervention was undertaken in eight children in total: four after the BB could not be retrieved endoscopically, and four without previous intervention. Three batteries were in the oesophagus, three in the stomach, one in the small bowel, and one battery had impacted above a pre-existing stricture in the rectum occurring secondary to a previous colorectal procedure. Importantly, one patient developed a life-threatening aorto-oesophageal fistula, which bled, and required surgical intervention. A second patient developed a tracheo-oesophageal fistula requiring surgical repair. After surgery, this child then developed an anastomotic leak requiring a prolonged stay in hospital and further interventions.

Median (i.q.r.) time to first intervention was 1.0 (0–1.0; range 0–60.0) days. A 1-year-old child with non-specific symptoms of feed intolerance underwent endoscopy at 60 days after developing vomiting.

Morbidities related to BB ingestion occurred in 14 (12.5 per cent) children at a median of 1.0 (0–10.0) days following ingestion. These included four (28.6 per cent) oesophageal and three (21.4 per cent) gastric ulcerations, and two (14.3 per cent) oesophageal strictures.

Median (i.q.r.) duration of hospital stay was 1.0 (0–2.0; range 1.0–70.0) days and was significantly longer in patients with an oesophageal BB on admission compared with BBs in other locations (4.5 *versus* 1.0 days, *P* < 0.001). Four patients with oesophageal BBs were admitted for 14 days or more. Follow-up was to a median (i.q.r.) of 1.0 (0–3.0; range 0.0–12.0) months.

### Mixed magnet and button battery ingestions

Five children ingested both magnets and BBs, at a median (i.q.r.) age of 14.8 (3.0–15.0) years. Three of these children were female, and two had an associated behavioural diagnosis. Three patients ingested a single BB alongside 23, six, and two magnets. One patient ingested two BBs and two magnets, and one patient ingested three BBs and two magnets. The foreign bodies were in the stomach (*n* = 3, 60.0 per cent), small intestine (*n* = 1, 20.0 per cent), or large intestine (*n* = 1, 20.0 per cent). All were diagnosed on plain-film imaging and the median (i.q.r.) number of plain-film X-rays was 5.0 (3.0–7.0; range 2.0–16.0). One patient underwent two CT scans.

One child required surgery for failure of the foreign bodies to progress. Two patients were managed endoscopically. The remaining two had successful conservative management. Median (i.q.r.) duration of hospital stay was 6.0 (3.0–11.0; range 1.0–30.0) days.

## Discussion

Magnet and BB ingestion requiring hospital admission is relatively frequent and is associated with a high burden of morbidity. More than one-third of children who ingest BBs undergo endoscopic treatment, and more than one-third who ingest multiple magnets undergo surgery. Two life-threatening complications of BB ingestion are described. This study highlights the need for further urgent action to prevent children accessing these potentially harmful objects. Multiple magnet ingestion frequently resulted in abdominal surgery, exposing children to not only short- and medium-term risks, such as anaesthetic complications and anastomotic leaks, but also the lifelong risks of subsequent complications, particularly adhesional bowel obstruction.

Key differences have been identified between children who ingest BBs and those who ingest magnets. Three-quarters of the children who ingested BBs were under the age of 6 years, suggesting that BBs are mainly swallowed by children who come across them accidentally within their play environment. Conversely, magnet ingestion is seen much more frequently in older children, including teenagers, demonstrating that children encounter magnets intentionally. This supports previous studies, where a median age of 6.8 years was observed in a UK cohort of 46 children ingesting magnetic foreign bodies. One study also observed that older girls may accidentally swallow magnets placed in their mouth to simulate piercings^4,14,15^. Children seem to more commonly ingest multiple magnets, suggesting that children have access to them in play in the form of toys. Unwitnessed ingestions, seen in younger children, have previously been shown to be associated with poorer outcomes, due to seeking medical attention late or presentations with non-specific clinical features^16^. In keeping with this, it is demonstrated that when multiple magnet ingestion occurs, younger age is associated with surgical intervention.

Children with behavioural diagnoses who ingested BBs were significantly older and ingested a significantly higher number of batteries, but a diagnosis of ASD or ADD was not associated with poorer outcomes. The higher proportion of older patients with behavioural diagnoses will be explained in part by the incidence of these diagnoses being higher in older children. However, this cohort of patients showed a higher-than-expected prevalence of ASD and ADD. The UK population prevalence of ASD and ADD in children and young people is 3 per cent^17^, whereas 8 per cent of children in this cohort had a behavioural diagnosis. This highlights the particular importance of limiting access to foreign bodies in this group of children.

The most common indication for surgical intervention following magnet ingestion was failure of the objects to progress on serial imaging. Progression of magnets through the gastrointestinal tract on imaging is reassuring, as static magnets suggest entrapment of bowel. Often however, when magnets were present in multiple parts of the gastrointestinal tract their radiological location was inconsistent with their location at intervention. As such, caution should be exercised when evaluating the radiological position of multiple ingested magnets.

There remains little evidence as to the optimum time interval between imaging studies, the findings that indicate that clinicians must intervene, and how soon intervention should occur after magnets stop progressing. The recent National Patient Safety Alert regarding ingestion of ‘super strong’ magnets advises repeat radiographs every 6–12 h after multiple magnet ingestion^18^. Without serial imaging it is difficult to track the movement of multiple magnets and identify those that have ceased to progress. A balance must be sought between limiting exposure to radiation while identifying the cases that require intervention. Previous studies advise surgical intervention if magnets have ceased to progress after 48 h^15^. However, given that almost 25 per cent of children undergoing surgery for magnet ingestion had bowel perforation, there is scope to recognize failure of magnet progression earlier^19^.

BBs cause caustic burns when opposed to the oesophageal mucosa, whereas more distal BBs are less likely to lie near the bowel wall and cause injury^20^. In a systematic review of 136 191 BB ingestions, only 8 per cent of complications resulted from BBs in the stomach and small intestine^8^. In the present study, BBs located in the stomach were either removed endoscopically or managed conservatively, with similar success rates. However, gastric mucosal damage was evident on gastroscopy in three children and treated with PPI therapy. A previous study has observed that 60 per cent had evidence of mucosal damage on endoscopy^21^. Current guidelines suggest that asymptomatic patients with BBs beyond the pylorus can be discharged with safety-netting advice^22^. The recently published European Society for Paediatric Gastroenterology, Hepatology and Nutrition guidelines state that asymptomatic children with BBs beyond the oesophagus require a follow-up X-ray after 7–14 days to check for passage, and endoscopic removal if impacted or symptomatic^20^.

In this study a single child developed a complication from a BB located beyond the pylorus. This patient had a previous history of surgically managed colorectal disease, and the battery was lodged above a stenotic segment of rectum. It is therefore important to undertake close observation and follow-up of patients presenting with an oesophageal BB with previous surgical history or an underlying gastrointestinal condition, and to provide thorough safety-netting advice to all children who ingest BBs.

This study is a large, contemporaneous, multicentre study that describes in detail the clinical course of children after magnet and BB ingestion. It gives an impression of the prevalence of the problem in the context of the current national measures in place, which are intended to limit children’s access to these potentially harmful objects.

The study is limited by the retrospective nature of the data collection, relying on complete, and accurate records in patient case notes. The use of ICD codes to identify patients may have missed cases of magnet and BB ingestion. Both factors create the potential for selection bias. A focus on paediatric surgical specialist centres, and only on those children admitted to hospital, means that the true incidence of magnet and BB ingestion, and the associated complication rate, is not described as only children who represent the most severe cases are represented here.

This study demonstrates that BB and magnet ingestions continue to occur and cause harm, despite strict product legislation around children’s toys. The proposed ‘Button Batteries (Safety) Bill’ is strongly supported by the findings of this study. Currently there is no legislation in the UK that controls the manufacture and sale of magnet products. All magnetic toys in the UK have a statutory requirement to be sold with a warning outlining the risks if swallowed or inhaled; however, this is often not disclosed at point of sale. In the USA, legislation regulating the sale of magnet products was passed in 2012, but this ruling was reversed in 2016 with an associated resurgence of magnet ingestion cases^14^. Action must be taken at a national and international level to regulate the sale of neodymium magnets, which have demonstrably caused significant harm to many children.

Currently there are no robust evidence-based consensus guidelines on the optimal imaging and management strategies for asymptomatic children following the ingestion of magnets. Detailed prospective studies are necessary to address this paucity of evidence. Children presenting to emergency departments, ear, nose and throat surgeons, paediatricians, and paediatric surgeons must be included and follow-up studies are necessary to identify how complications manifest in the long-term. The management of children swallowing magnets and BBs should be audited against best treatment guidelines.

## Organisation and Writing Group

Jonathan J. Neville (University Surgery Unit, Faculty of Medicine, University of Southampton, Southampton, UK), Rachel Harwood (Department of Paediatric Surgery, Alder Hey Children’s Hospital, Liverpool, UK), George S. Bethell (University Surgery Unit, Faculty of Medicine, University of Southampton, Southampton, UK), Hannah Rhodes (University Surgery Unit, Faculty of Medicine, University of Southampton, Southampton, UK), Felicity Arthur (Department of Paediatric Surgery, Royal Hospital for Children, Glasgow, UK), Mary P. Eastwood (Department of Paediatric Surgery, Royal Belfast Hospital for Sick Children, Belfast, UK), Sesi Hotonu (Department of Paediatric Surgery, Edinburgh Royal Infirmary, Edinburgh, UK), Lucinda Tullie (Specialist Neonatal and Paediatric Surgery, Great Ormond Street Hospital NHS Foundation Trust, London, UK) and Nigel J. Hall (University Surgery Unit, Faculty of Medicine, University of Southampton, Southampton, UK).

## Collaborators

R. Coulson (Royal Belfast Hospital for Sick Children, Belfast, Northern Ireland), S. Lawther (Royal Belfast Hospital for Sick Children, Belfast, Northern Ireland), K. Burns (Bristol Royal Hospital for Children, Belfast, UK), C. S. Chacon (Chelsea Children's Hospital, London, UK), T. Boam (Chelsea Children's Hospital, London, UK), S. A. Clarke (Chelsea Children's Hospital, London, UK), J. Hallett (Edinburgh Royal Infirmary, Edinburgh, UK), N. Valliant (Evelina Children's Hospital, London, UK), T. Hemanshoo (Evelina Children's Hospital, London, UK), A. Tahira (Royal Hospital for Children, Glasgow, UK), E. Decker (Alder Hey Children's Hospital, Liverpool, UK), T. Ahmed (Norfolk and Norwich University Hospital NHS Trust, Norwich, UK), J. Cave (Norfolk and Norwich University Hospital NHS Trust, Norwich, UK), A. Ram (Norfolk and Norwich University Hospital NHS Trust, Norwich, UK), M. Shenoy (Nottingham Children's Hospital, Nottingham, UK), M. John (Nottingham Children's Hospital, Nottingham, UK), M. Wyn (Nottingham Children's Hospital, Nottingham, UK), L. Wilkins (John Radcliffe Hospital, Oxford, UK), B. Allin (John Radcliffe Hospital, Oxford, UK), A. Fagelnor (John Radcliffe Hospital, Oxford, UK), G. Bough (The Royal London Hospital, London, UK), A. T. Mohd-Amin (The Royal London Hospital, London, UK), and R. Trenear (The Royal London Hospital, London, UK).

## Funding

The authors have no funding to declare.

## Data Availability

Any reasonable requests to share data will be considered by the PSTRN committee, subject to institutional agreements and ethical approval. Data requests should be sent to the corresponding author.
